# Cloning and Characterization of a Novel Alginate Lyase from *Paenibacillus* sp. LJ-23

**DOI:** 10.3390/md20010066

**Published:** 2022-01-12

**Authors:** Mingpeng Wang, Lei Chen, Zhengyu Lou, Xueting Yuan, Guiping Pan, Xiaoyan Ren, Pengyu Wang

**Affiliations:** College of Life Science, Qufu Normal University, Qufu 273100, China; qsdwmp2018@qfnu.edu.cn (M.W.); lwlzy03@163.com (Z.L.); nulixuexi0902@163.com (X.Y.); pgp19553710386@163.com (G.P.); bkk18854782484@163.com (X.R.); wpy2569909304@163.com (P.W.)

**Keywords:** alginate lyase, *Paenibacillus*, alginate oligosaccharides, alginate degradation, drug delivery

## Abstract

As a low molecular weight alginate, alginate oligosaccharides (AOS) exhibit improved water solubility, better bioavailability, and comprehensive health benefits. In addition, their biocompatibility, biodegradability, non-toxicity, non-immunogenicity, and gelling capability make them an excellent biomaterial with a dual curative effect when applied in a drug delivery system. In this paper, a novel alginate lyase, Algpt, was cloned and characterized from a marine bacterium, *Paenibacillus* sp. LJ-23. The purified enzyme was composed of 387 amino acid residues, and had a molecular weight of 42.8 kDa. The optimal pH of Algpt was 7.0 and the optimal temperature was 45 °C. The analysis of the conserved domain and the prediction of the three-dimensional structure indicated that Algpt was a novel alginate lyase. The dominant degradation products of Algpt on alginate were AOS dimer to octamer, depending on the incubation time, which demonstrated that Algpt degraded alginate in an endolytic manner. In addition, Algpt was a salt-independent and thermo-tolerant alginate lyase. Its high stability and wide adaptability endow Algpt with great application potential for the efficient preparation of AOS with different sizes and AOS-based products.

## 1. Introduction

As a linear acidic polysaccharide, alginate is one of the main skeleton components in the matrix and cell walls of brown seaweed, and maintains a stable cell structure [1,2]. It consists of α-L-guluronic acid (G) and β-D-mannuronic acid (M), which are its constituent monomers. Two kinds of constituent monomers are linked by 1,4-O-glycoside bonds. There are three types of constituent fragments in the long chain of alginate, including poly-L-guluronate (poly G), poly-D-mannuronate (poly M), and the heteropolyuronic blocks (poly MG) [3]. The overall composition of the two uronic acids, the ratio of G to M, and their distribution along the polymer chain are varied and depend on the species, habitats, and harvesting season of algae. As a natural polysaccharide, alginate has been widely applied in food, cosmetic, and biomedical industries due to its chelation, gelation, and hydrophilic properties [4,5]. In biomedicine, alginate is mainly used as a matrix or carrier to realize the sustained release and enhanced efficacy of a drug in targeted or localized drug delivery systems. However, its polymeric structure, poor solubility, and low bioavailability have greatly limited the biomedical application of alginate as a direct effector or functional component [6].

Alginate can be degraded enzymatically or chemically, generating alginate oligosaccharides (AOS), which have lower viscosity and smaller sizes. Compared with alginate, AOS have better solubility and improved bioavailability [7]. As a consequence, increasing attention has been paid to AOS due to their better beneficial effects and pharmacological activities in biomedicine. Growing evidence suggests that, when applied as signal molecules and prebiotics, AOS could improve antitumor immune efficacy [8], regulate whole-body metabolism, and recondition and remodel gut microbiota. Recently, research has indicated that AOS could also be used as drug carriers in the development of novel drug delivery systems. AOS rich in M components were used as matrices for posaconazole (POS) delivery, intensifying its antifungal activity [9]. The spherical gel of AOS-containing lysosomes could maintain the activities of lysosomes and prolong its release [10]. A novel delivery system based on zein-AOS complex nanoparticles was reported as a carrier of curcumin [11]. The delivery system showed an excellent sustained release effect and acid resistance, protecting curcumin from digestion by gastric juices. A bi-functional AOS–polymyxin conjugate was developed to improve the effectiveness of antibiotics in the treatment of infections induced by multidrug-resistant Gram-negative bacteria [12]. The addition of the AOS component directly reduced the cytotoxicity of the initial antibiotic whilst retaining the antimicrobial and antibiofilm activities. Therefore, AOS, as a natural and burgeoning drug carrier, exhibited a dual curative effect due to its intrinsic multiple biological activities, giving AOS broad application prospects.

Alginate lyases are crucial and efficient tools for preparing AOS for various specific biological activities [3,13]. However, the low enzymatic activity, narrow environmental adaptability, weak biochemical stability, and high-cost of manufacture of existing enzymes still restrict the industrialization and commercialization of AOS-based products [14]. Thus, it is vital to continually discover and obtain novel alginate lyases with excellent properties from diverse sources [15,16]. So far, numerous alginate lyases have been purified and identified from many kinds of microorganisms. Based on the CAZy database, alginate lyases are grouped into 12 polysaccharide lyase families (PLs): PL-5, PL-6, PL-7, PL-14, PL-15, PL-17, PL-18, PL-31, PL-32, PL-34, PL-36, and PL-39 [17,18]. Alginate lyases that belong to different PL families have diversified structures and action modes [3,19]. As for the PL-6 family, most lyase members usually display a parallel β-helix fold in three dimensions [3,19]. Compared to members of other PL families, the alginate lyases of the PL-6 and PL-31 families had been discovered relatively seldomly, and the substrate recognition and cleavage mechanism of most PL-6 alginate lyases remain unclear.

In a previous study, using alginate as the sole carbon source, we screened and isolated the microbes from the surface of three different kinds of seaweeds, and finally obtained 12 alginate lyase-producing strains. The alginate lyase excreted by the LJ-23 strain showed the second-highest enzymatic activity among these strains. Based on the 16S rRNA gene (accession no. KX959965) sequencing, it was suggested that the LJ-23 strain belonged to *Paenibacillus taichungensis*, with a sequence similarity of 99.25%. The analysis of the neighbour-joining phylogenetic tree further verified the identification of the LJ-23 strain. Therefore, it was classified as part of the genus *Paenibacillus* and nominated as *Paenibacillus* sp. LJ-23. In addition, the genus *Paenibacillus* has been reported to show excellent capacity at degrading a variety of polysaccharides with complex structures, including chitin, xylan, cellulose, alginate, and even the capsular polysaccharide from bacteria [16,20,21,22,23,24,25]. However, the gene encoding alginate lyase from the genus *Paenibacillus* has rarely been identified. An alginate lyase of *Paenibacillus* sp. str. FPU-7, isolated from soil, were purified and identified as a novel alginate lyase that belonged to the PL-31 family [26].

Here, we cloned a novel alginate lyase, Algpt, from a marine bacterium, *Paenibacillus* sp. LJ-23, which is drastically different from terrestrial bacterium and the gut microbe of molluscs, indicating that Algpt might has unique features. Thus, we studied its enzymatic characteristics and further investigated its degradation products and three-dimensional structure.

## 2. Results and Discussions

### 2.1. Cloning and Sequence Analysis of Algpt

There are four genome assembly and annotation reports of *P. taichungensis* in the NCBI Genome Project (http://www.ncbi.nlm.nih.gov/genome/, Accessed date: 28 December 2021). However, the gene annotated as alginate lyase, poly-α-L-guluronate lyase, poly-β-D-mannuronate lyases, or other associated names was not found. According to the homology of the amino acid sequence, alginate lyases could be grouped into different families of polysaccharide lyases (PL): PL-5, P-6, PL-7, PL-14, PL-15, PL-17, and PL-18 [17,18]. They usually have diverse three-dimensional structures, including a parallel β-helix fold, a (α/α)_n_ toroid fold, and a β-jelly roll fold. The alginate lyases from the PL-6 and PL-31 families have a β-helix fold, while the PL-5, PL-15, and PL-17 members usually have (α/α)_n_ toroid folds [3,19]. Despite their distinct enzymatic properties, most of the alginate lyases from the PL-7 family share a typical structure consisting of a β-jelly roll fold that forms a catalytic crack and three adjacent β-sheets that contain catalytic amino acid residues [27,28]. The conserved domains of the PL14 and PL18 members also appeared in the form of a β-jelly roll fold. Accordingly, we tried to search the genes or proteins that were associated with these special structural features. Two genes, of which the product was annotated as a parallel beta-helix pectate lyase-like protein, were found in the *Paenibacillus taichungensis* strain DB-4 genome. Based on their gene sequences, the primers were designed for putative alginate lyase gene cloning.

**Figure 1:**
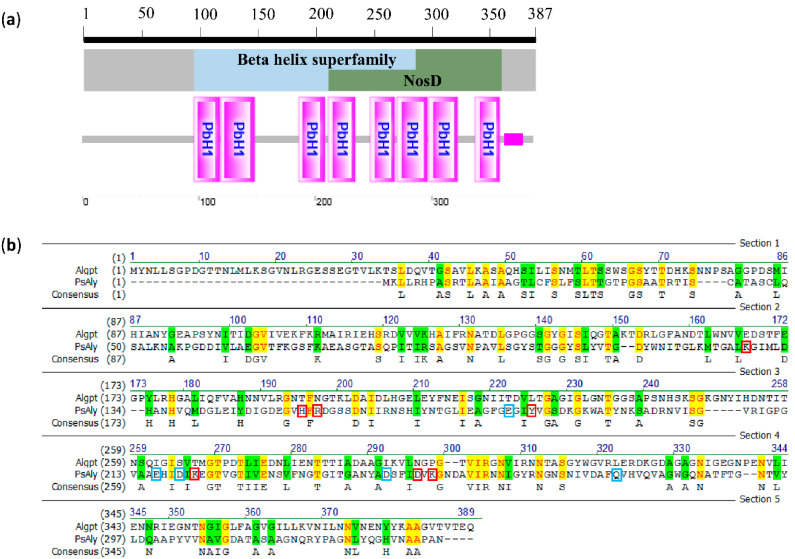
Sequence analysis and comparison of Algpt. (**a**) Conserved region analysis. Algpt contains two overlapping domains, a β-helix domain and a NosD domain. PbH1 means one parallel β-helix repeat. (**b**) Pairwise sequence alignment analysis of Algpt and a PL-31 alginate lyase, PsAly. Identical amino acid residues are shaded in yellow, and similar amino acid residues are shaded in green. Weak similar amino acid residues are shown in green characters. The catalytic sites of PsAly are marked with red boxes. Residues associated with the substrate or cation binding are labelled with cyan boxes.

Fortunately, one positive clone, which showed a prominent alginate degradation zone on the screening plate, was acquired. A putative gene encoding alginate lyase, nominated as *algpt*, was identified by sequencing. The length of the *algpt* gene is 1161 bp. Its protein product, named Algpt, was composed of 387 amino acids with a calculated molecular weight of 40873.47 Da. The amino acid sequence of Algpt has the highest sequence identity (54%) with a parallel β-helix repeat protein-containing pectate lyase domain from *Neobacillus niacini* AT2.8 (NYE07960.1), excluding the genus *Paenibacillus.* The domain architecture analysis suggested that Algpt should be classified into the β-helix superfamily (cl37851), as shown in Figure 1a. The feature of the β-helix fold indicated that Algpt belonged to the PL-6 or the PL-31 family. First, we compared the amino acid sequence of Algpt with PsAly, a PL-31 alginate lyase from the genus *Paenibacillus*. As shown in Figure 1b, the sequence similarity between Algpt and PsAly is less than 30%, and the sequence identity is 13.9%. Moreover, the substrate-binding sites and catalytic sites of PsAly, which are vital to enzymatic activity, were not matched to the Algpt sequence. This result showed that Algpt was significantly different from the PsAly from the PL-31 family in terms of the primary protein sequence, despite the fact that both belong to the same genus *Paenibacillus* and share the β-helix structure.

**Figure 2:**
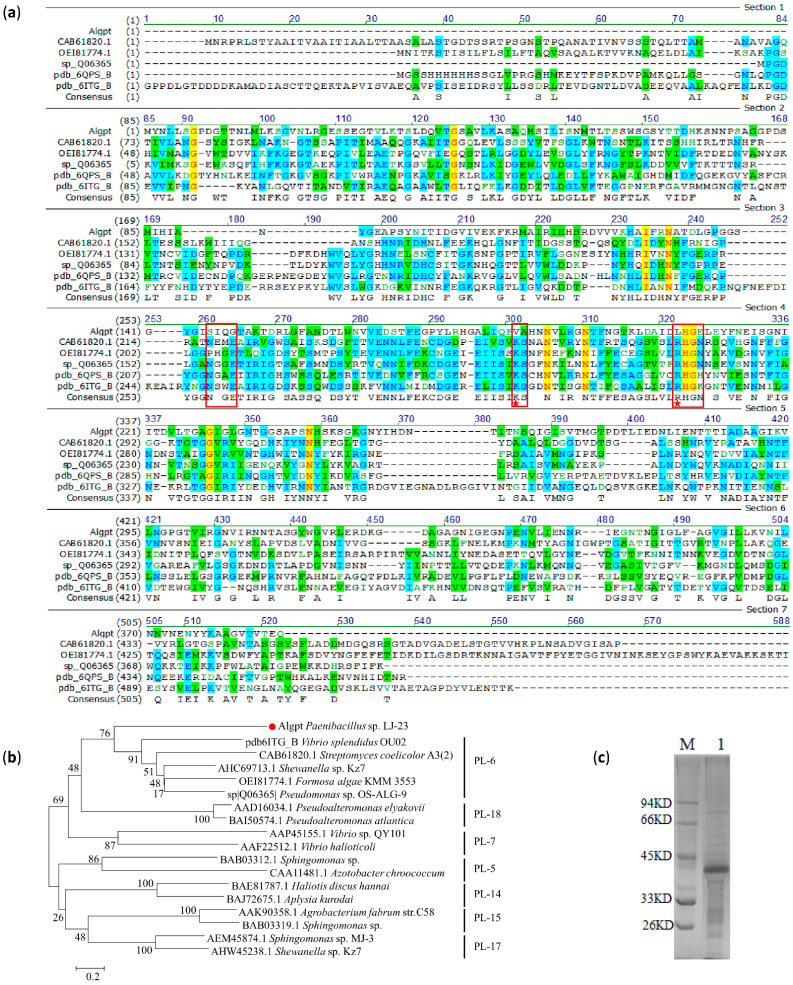
Sequence comparison, phylogenetic relationship, and purification of Algpt. (**a**) Multiple amino acid sequence alignment analysis of conversation domain of Algpt and different PL-6 alginate lyases. Identical amino acid residues are shaded in yellow, conservative amino acid residues are shaded in cyan, and similar amino acid residues are shaded in green. Weak similar amino acid residues are shown in green characters. Three conserved regions of the PL-6 alginate lyase are marked with red boxes. Residues associated with catalysis are labelled with red stars. OUC-ScCD6 (CAB61820.1) from *Streptomyces ecolicolor* A3(2), ALFA4 (OEI81774.1) from *Formosa algae* KMM 3553, AlyP(SP ID: Q06365) from *Pseudomonas* sp. OS-ALG-9, *Bcel*PL6 (PDB ID: 6QPS_B) from *Bacteroides cellulosilyticus* CRE21, AlyF (PDB ID: 6ITG_B) from *Vibrio splendidus* OU02. (**b**) Neighbour-joining tree for the amino acid sequence of Algpt and other PL-6 alginate lyases. (**c**) SDS-PAGE analysis of purified Algpt. Lane M, protein standard marker (26–94 kDa); lane 1, purified Algpt.

The alginate lyase of the PL-6 family had three conserved domains, “NG(G/A)E”, “(I/V)KS”, and “R(H/S)GN”, which contribute to the substrate binding and catalytic activity [29]. Based on further analysis of the multi-sequence alignment result, we discovered that Algpt contains the “R(H/S)GN” region, which is highly conserved in the PL-6 alginate lyases [17,30] (Figure 2a). However, the “(I/V)KS” region was not found in the Algpt sequence, and the amino acid residues of Algpt corresponding to “NG(G/A)E” region showed weak similarity with other PL-6 alginate lyases. Although the amino acid sequence of Algpt has a low sequence similarity (29.1%) to PL-6 alginate lyase, the phylogenetic tree result showed that the Algpt sequence was closely related to the members of the PL-6 family (Figure 2b). Taken together, the protein sequence analysis indicated that Algpt might be a novel alginate lyase which folds into a β-helix structure similar to that of the PL-6 and PL-31 families and exhibits a significantly different primary sequence conservation from the members of PL-6 and PL-31 families.

Subsequently, the recombinant plasmid pET21a-Algpt was transferred into *E. coli* BL21 (DE3) and expressed as a fusion protein with a C-terminal His tag. After 12 h of IPTG induction, the recombinant Algpt was purified using Ni–NTA resin and showed a clear band around 42 kDa in SDS-PAGE gel (Figure 2c), which is basically in accordance with the predicted molecular weight (40873.47 Da) of naked Algpt, considering the mass increase caused by the His tag. As for the PL-6 alginate lyases, the molecular weight varied from 47.8 kDa to 107 kDa [31,32]. Obviously, the molecular weight of Algpt, identified in this study, is smaller than that of the reported members of the PL-6 alginate lyases.

### 2.2. Biochemical Properties of Algpt

**Figure 3:**
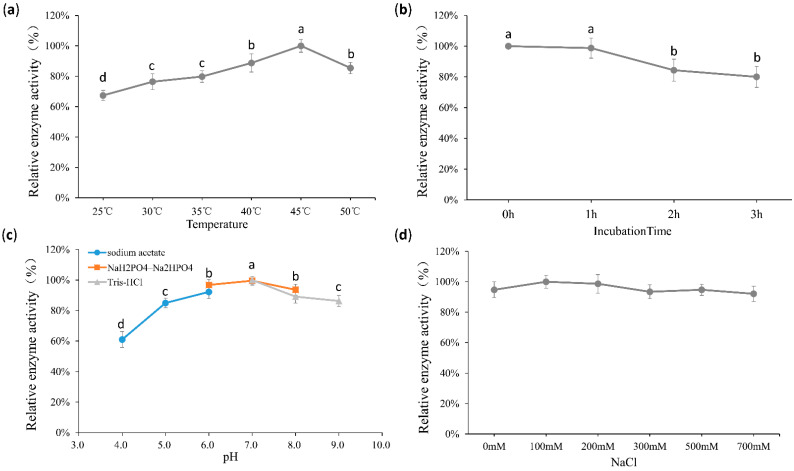
Enzymatic features of the recombinant Algpt. (**a**) The effect of temperature on Algpt activity. Activity at 45 °C was taken as 100%. (**b**) The temperature stability of Algpt. The reaction mixtures were pre-incubated at 50 °C for different hours. Then, the remnant activities were tested. The activity of treatment without pre-incubation was taken as 100%. (**c**) The optimal pH of Algpt. Here, 50 mM buffer systems of sodium acetate (pH 4.0–6.0), NaH_2_PO_4_–Na_2_HPO_4_ (pH 6.0–8.0), and Tris-HCl (pH 7.0–9.0) were applied to achieve the required pH value. Activity at pH 7.0 was taken as 100%. (**d**) The effect of NaCl on Algpt activity. The reaction mixture without NaCl was taken as 100%. Each value represents the mean of three replicates ± standard deviation. Mean values (*n* = 3) with different letters are significantly different (*p* < 0.05) according to Duncan’s multiple range test.

The enzymatic characteristics of Algpt were further studied. The optimum temperature of the recombinant Algpt was 45 °C (Figure 3a), and the lyase still retained over 80% of its activities after incubation at 50 °C, with the time of incubation ranging from 1 h to 3 h (Figure 3b). Based on the comprehensive analysis of the reaction temperature and the thermostability results, Algpt was considered to be an enzyme with a wide temperature tolerance, and it was efficient and stable at 30–50 °C. The optimal pH of Algpt was pH 7.0, and it showed high activity in the range of pH 5.0–9.0 (Figure 3c). As shown in Figure 3d, there was almost no difference in enzymatic activity whether NaCl existed or not, indicating that NaCl was not the critical factor affecting enzymatic activity.

According to previously published reports, most alginate lyases exhibited the highest enzymatic activity in conditions of 30 °C–50 °C and pH 7.0–9.0. As shown in Table 1, a PL-7 alginate lyase, Alg2A, was confirmed to show optimum activity at 40 °C and pH 8.5, while the highest activity of another PL-7 member, Algb, was achieved at 30 °C and pH 8.0. As for PL-6 family alginate lyases, AlyPL6, OUC-ScCD6, and FsAlyPL6 all showed optimal activity at or over 45 °C, while AlyF and BcelPL6 have the optimum catalysis temperature of 30 °C. Significantly, Alg2A, Algb, AlyF, and FsAlyPL6 exhibited a relatively narrow temperature range for retaining 80% of their enzymatic activity, while AlyPL6, OUC-ScCD6, and Algpt could maintain high enzymatic activity across a broader temperature range. As for pH, AlyPL6, OUC-ScCD6, and FsAlyPL6 were memorable due to a higher optimal reaction pH value. However, once the pH value of the reaction system fell below 7.0, their enzymatic activity was significantly reduced, and they even lost 60% of their activity. Our results showed that Algpt still retained over 80% of its enzymatic activity at pH 6.0. As the pH went down, around 80% of its activity was maintained at pH 5.0, or 60% of its activity at pH 4.0 (Figure 3c). Furthermore, Algpt has no special requirement for NaCl due to its maintenance of high enzymatic activity with and without NaCl. Thus, Algpt is relatively widely adaptable to broad thermal, pH, and salinity ranges, showing an advantage in actual production under changeable reaction conditions and a great potential to be applied in the seaweed processing industry.

### 2.3. Analysis of the Enzymatic Hydrolysate

**Figure 4:**
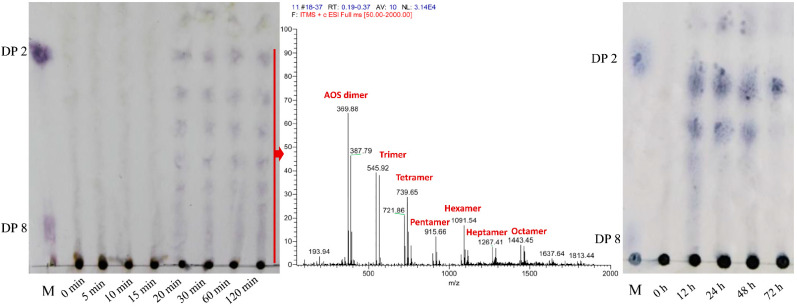
TLC and ESI–MS analysis of degrading products of Algpt towards alginate. The ESI–MS profile was the result of products from the sample incubation for 120 min.

Thin layer chromatography (TLC) and electrospray ionization mass spectrometry (ESI–MS) were applied to analyse the enzymatic hydrolysate of the alginate substrate at different reaction times. As shown in Figure 4, the alginate substrate was rapidly degraded by Algpt at the early stage of enzymatic hydrolysis. The hydrolysis products mainly contained AOS with various low degrees of polymerization (DPs 2–8). The molecular mass of the AOS product at a reaction time of 120 min was subsequently verified via ESI-MS, and the result was consistent with TLC. As the proceeding of hydrolysis continued, the oligosaccharide dimers, trimers, and tetramers accounted for a larger fraction at an incubation time of 12 h, while dimers were the dominant hydrolysis products after 72 h. The results above indicate that Algpt is an endolytic lyase.

Several action modes and types of end products appeared in different PL6 family alginate lyases. Most of PL-6 family members are endolytic alginate lyases, such as *Bcel*PL6, AlyF, AlyMG, and Alg823, generating AOS with DP2, DP3, DP2-3, and DP2-6 as the major degradation products, respectively [17,18,31,37]. As for other PL-6 alginate lyases, the OalC6 from *Cellulophaga* sp. SY116, OalS6 from *Shewanella* sp. Kz7, and Pedsa0631 and Patl3640 from *Pseudoalteromonas atlantica* T6c could degrade polyG or alginate polymer in an exolytic manner to produce monosaccharides [32,38,39]. Although the specific degradation process of the polysaccharide substrate and the detailed action modes of most PL-6 family alginate lyase, including Algpt, have not been fully described, our results suggested that Algpt may be a potential tool to prepare AOS with different DPs by precisely controlling the incubation time and the enzyme-to-substrate ratio. The oligosaccharide (DP > 8) appeared at the initial stage of the degradation process and may be applied in the development of a novel drug carrier. Several reports suggested that AOS with higher polymerization could play a dual role (effector and carrier) in drug delivery systems, showing obvious advantages compared with alginate-based carriers [9,10,11,12]. AOS with lower DPs (2–4) could be used in the field of agricultural production as plant stimulants to promote their growth and development [40,41,42,43], or in the field of biomedicine as signalling molecules to regulate body function [8,44,45]. An AOS mixture with DP 2–8 isolated from the middle stage of hydrolysis reaction (1–4 h) could be used to develop a series of single AOS standards.

### 2.4. Three-Dimensional Structure Comparison and Prediction of Novel Enzyme

**Figure 5:**
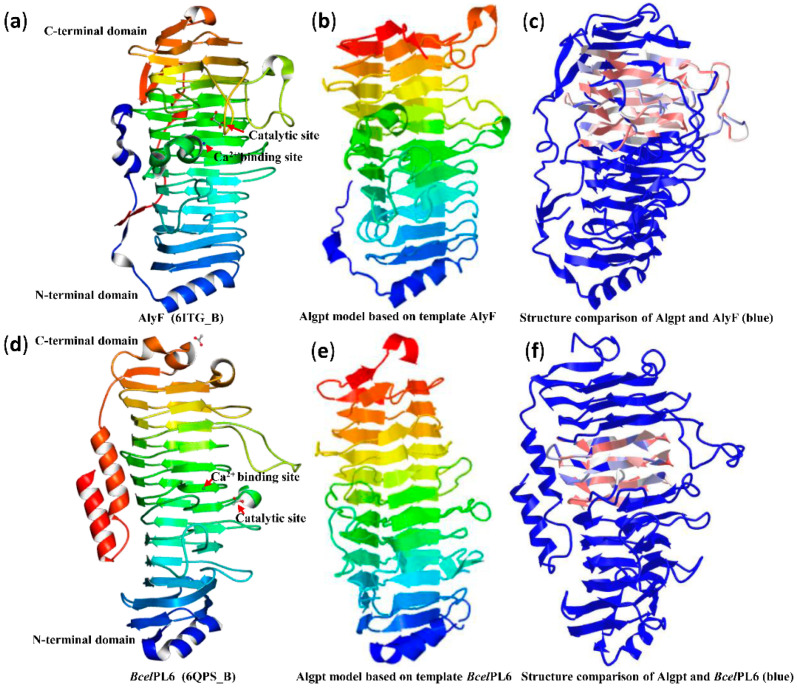
Predicted structure of Algpt and its comparison with AlyF and *Bcel*PL6. (**a**) The structure of AlyF with 536 amino acids (Gly1-Lys536). (**b**) The structure of Algpt based on AlyF as a template. (**c**) Structural comparison of Algpt and AlyF. The structure of AlyF is shown in blue. The well-matching conserved domain between Algpt (Ile204-Asn371) and AlyF (Ile260-Asn432) is shown in a mixed colour (light pink, light white, light blue). (**d**) The structure of *Bcel*PL6 with 467 amino acids (Met1-Arg467). (**e**) The structure of Algpt based on *Bcel*PL6 as a template. (**f**) Structural comparison of Algpt and *Bcel*PL6. The structure of *Bcel*PL6 is shown in blue. The well-matching conserved domain between Algpt (Val165-Lys245) and *Bcel*PL6 (Ile230-Lys308) is shown in a mixed colour (light pink, light white, light blue).

According to the homologous structure of the alginate lyases from *Vibrio splendidus* OU02 (PDB ID: 6ITG_B) and *Bacteroides cellulosilyticus* CRE21 (PDB ID: 6QPS_B), the three-dimensional models of Algpt based on different templates were constructed using Phyre2 [18,35]. Although the sequence identity (15%) with both templates was relatively low, the protein models of Algpt were successfully constructed with 99.7% and 99.69% confidence, respectively, because they share the same parallel β-helix fold. However, using another alginate lyase belonging to the PL-31 family, which also has a β-helix fold as a template (PDB ID: C6KFN_A), the 3D structure of Algpt was not successfully modelled due to low match confidence between our sequence and this template [26]. As shown in Figure 5b,e, the structures of two putative Algpt based on different templates were both speculated to form a “spring-like” structure with several parallel β-helixes. Further structure comparison suggested that Algpt shared a similar β-helix structure with the C-terminal domain of AlyF (Figure 5c), or the middle domain of *Bcel*PL6 (Figure 5f), but was poorly matched with the N-terminal domain of both templates. The part of Algpt protein that matches its two templates just right covers the catalytic sites and ion binding sites of the templates (Figure 5a,c,d,f). Especially in the *Bcel*PL6 sequence, His-271 was also essential for the catalytic activity, besides generally conserving Lys-249 and Arg-270 [35]. Combined with the analysis of multiple amino acid alignments (Figure 2a), these results indicated that the three residues “RHG” (Arg-177, His-178, and Gly-179) and “LHG” (Leu-206, His-207 and Gly-208) might be two putative catalytic regions in the Algpt sequence.

**Figure 6:**
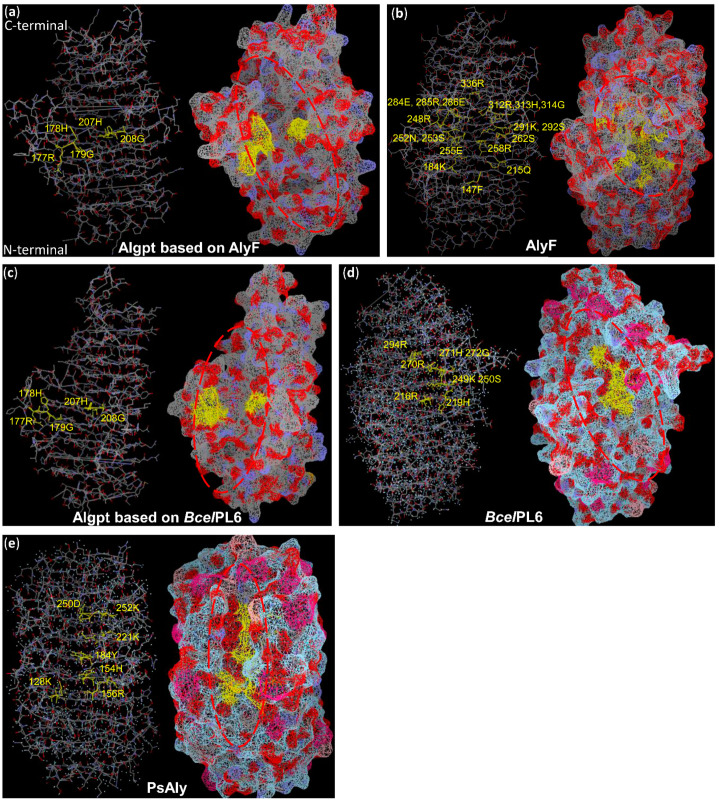
The structural comparison between Algpt and other alginate lyases with a β-helix fold (*Bcel*PL6 and AlyF from the PL-6 family, PsAly from the PL-31 family). The 3D models of different alginate lyase displayed in backbone and surface style, including (**a**) putative Algpt based on AlyF, (**b**) AlyF, (**c**) putative Algpt based on *Bcel*PL6, (**d**) *Bcel*PL6, (**e**) PsAly. The catalytic sites, cation binding sites, and substrate docking sites are marked in yellow. Active clefts are surrounded by red circles. The cleft of *Bcel*PL6 and PsAly are in an open-ended form, while the cleft structure of the PL-6 alginate lyase, AlyF, is a semi-closed form. The models were constructed using Vector-NTI software, based on the PDB data of each protein.

In addition, three-dimensional models of PL-6 and PL-31 alginate lyases showed that the sites related to enzyme function, including catalytic sites, ion binding sites, and substrate coordination sites, were spatially close enough to each other to form the active cleft that could dock with the substrate and perform the degradation function (Figure 6b,d,e). Although the active cleft structures varied in different alginate lyases, such as having an open-ended form or a semi-closed form, the functional sites were distributed around the active cleft in a hierarchical or confrontational arrangement on the β-helix structure (Figure 6b,d,e). In the two structure models of Algpt based on different templates, the active cleft was an open-ended form, and the two putative functional regions, “RHG” and “LHG”, were right on the edge of the cleft in a confrontational arrangement (Figure 6a,c). These predictions, based on existing data, still give us valuable information. Based on this three-dimensional structure model, we could search and locate the amino acid residues that distributed in the active cleavage and arranged regularly, and verify the relationship between them and enzymatic activity using a site-directed mutation technique.

**Figure 7:**
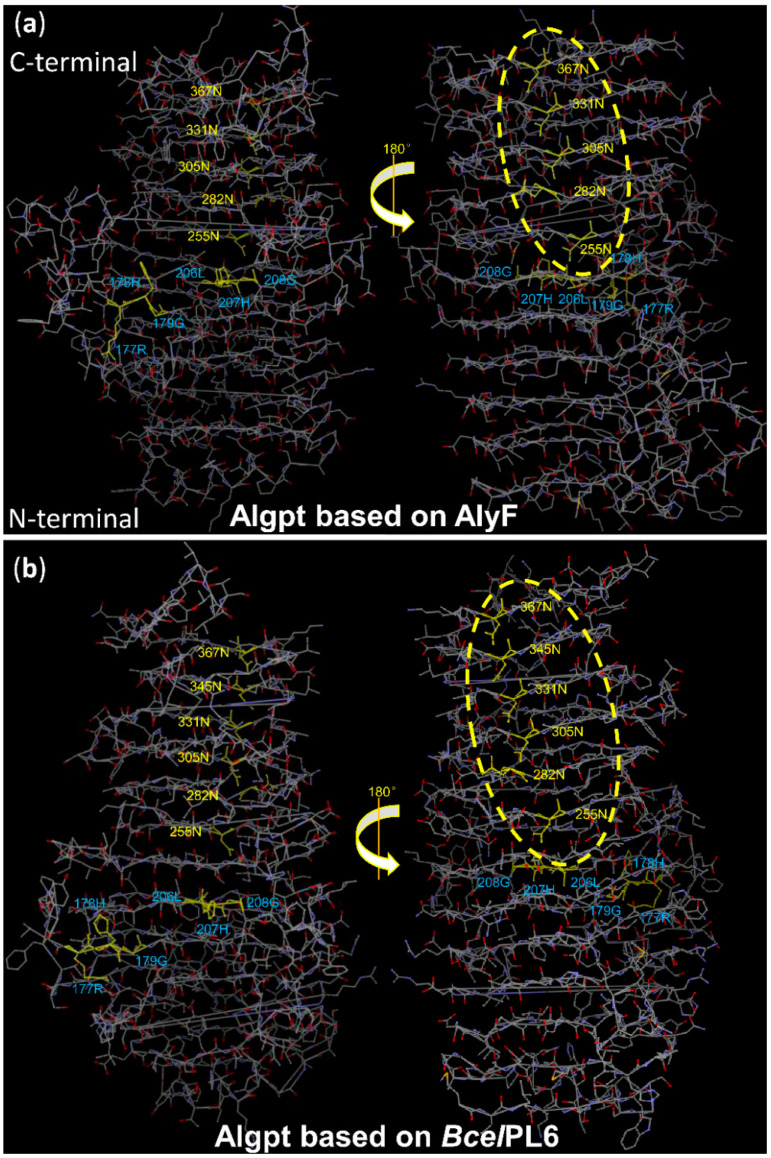
Asparagine ladder of the β-stranded coils in the predicted Algpt models. The model of Algpt based on (**a**) AlyF and (**b**) *Bcel*PL6 with the putative asparagine ladder and catalytic sites as stick models showing Asn (yellow) and showing Arg, His, Gly, and Leu (blue), respectively; asparagine ladders are marked with a yellow circle.

The asparagine ladder is a typical conserved structure in members of the PL-1, -6, and -9 families, with β-helix folds [46,47,48]. A sequence analysis of the alginate lyase from the PL-6 and PL-31 families revealed that the number of the asparagine residues in asparagine ladders are basically constant, indicating that they are vital for the folding and stability of the β-helix structure. Remarkably, conserved asparagine ladders are only found in β-helix alginate lyases, but not in pectate lyases [18]. Asparagine ladders may provide strong support for the conformational stability of the alginate lyase during its interaction with the polyelectrolyte alginate [18]. As shown in Figure 7, both predicted Algpt models exhibited asparagine ladders with five or six -“steps”, respectively, which spanned almost the half-length of the β-helix and were located at the opposite side of the active cleft. This result confirmed its identity as an alginate lyase and certainty its β-helix structure.

The putative three-dimensional structure and catalytic site of Algpt were partly similar to those of the PL-6 family members, but there are still noticeable differences in its primary sequence and enzymatic features compared with the existing PL-6 and PL-31 alginate lyase. Therefore, Algpt might be classified as a novel alginate lyase, not belonging to the PL-6 or PL-31 families.

## 3. Conclusions

In summary, we successfully identified and cloned a new alginate lyase encoding gene from marine bacteria strain *Paenibacillus* sp. LJ-23 by mining available sequence information and structural associations. The molecular mass of the recombinant Algpt is 42.8 kDa, which is lower than that of any of the PL-6 family alginate lyases previously reported. The purified enzyme showed the maximum activity at 45 °C and pH 7.0. We predicted the structure of Algpt, which displays a typical structure of a β-helix fold normally found in PL-6 alginate lyases. Algpt was a salt-independent alginate lyase with wide temperature tolerance and pH adaption. Depending on the reaction conditions, Algpt could generate oligosaccharides with distinct a DP (2–4, 2–8, or >8) using alginate as a substrate. Our study indicated that Algpt might be a potentially powerful tool for producing AOS and AOS-based biomaterials with diverse structures and functions for application in different fields.

## 4. Materials and Methods

### 4.1. Materials

Sodium alginate was purchased from Sinopharm Chemical Reagent Co., Ltd. (Shanghai, China). Standard alginate oligosaccharides, from disaccharide to octasaccharide, were purchased from QINGDAO HEHAI BIOTECH Co., Ltd. (Qingdao, China). Ni-NTA His·Bind Resin was purchased from EMD Millipore Corp (Burlington, MA, USA). EasyPfu DNA polymerase and EasyPure genomic DNA kit were purchased from Transgen Biotech (Beijing, China). Other chemicals and reagents used in this study were of analytical grade.

### 4.2. Screening and Identification of Strain LJ-23

Three kinds of brown seaweeds were collected from the coast of Nanhuangcheng Island, China. The treatment of seaweeds was carried out as described in our previous study [16]. The ALG medium containing 5 g/L (NH_4_)_2_SO_4_, 1 g/L MgSO_4_, 2 g/L K_2_HPO_4_, 5 g/L sodium alginate and 20 g/L agar (pH 7.2–7.4) was used for the screening of alginate lyase-excreting strains. After Gram’s iodine staining, the clear zone around the strain colony indicated that the strain secreted alginate lyase into the medium. The strains with a clear zone were isolated and these strains were selected for further analysis. To identify the LJ-23 strain, the 16S rRNA gene was amplified through PCR by using primers 27F (5′-AGAGTTTGATCCTGGCTCAG-3′) and 1492R (5′-TACGGTTACCTTGTTACGACTT-3′). The purified PCR fragment was sequenced and compared with the reported 16s rRNA sequences in GenBank by using BLAST. A phylogenetic tree was constructed using the neighbour-joining method and the Kimura two-parameter model in the MEGA 5.1 program.

### 4.3. Sequence Analysis of Algpt

The Algpt protein sequence was translated based on the sequence information of its encoding gene, while the other alginate lyase sequences were found in the database according to the related literature. Both the sequence analysis and structure prediction were achieved using different online tools. The sequence similarity of Algpt was analysed using NCBI BLAST and the protein function domains were identified using NCBI CDD and SMART (http://smart.embl-heidelberg.de/, Accessed date: 28 December 2021). Multiple amino acid sequence alignment was accomplished using Clustal Omega (http://www.clustal.org/omega/, Accessed date: 28 December 2021) and Vector-NTI software (Invitrogen, Thermo Scientific, Waltham, MA, USA). The Phyre2 Server (http://www.sbg.bio.ic.ac.uk/phyre2, Accessed date: 28 December 2021) was applied to construct the three-dimensional structure of Algpt based on different templates. The comparison of the predicted three-dimensional structure of Algpt with different templates was achieved using the NCBI Structure Tool, iCn3D: web-based 3D structure viewer (https://structure.ncbi.nlm.nih.gov/Structure/icn3d/, Accessed date: 28 December 2021).

### 4.4. Cloning and Recombinant Expression of the Alginate Lyase from Paenibacillus sp. LJ-23

The primers for cloning the alginate lyase gene were designed based on the sequence of a suspected gene (DEU73_101198) from *P*. *taichungensis* DB-4, which encoded a parallel beta-helix pectate lyase-like protein. The forward primer-containing restriction site Nde I was designed as 5′-GGAATTCCATATGTACAATTTGTTGTCCGG-3′ and the reverse primer containing restriction site Xho I was designed as 5′-CCGCTCGAGCTGCTCGGTAACGGTAAC-3′. The nucleotide fragments amplified via PCR were purified and ligated to pET-21a(+) expression vector (Novagen, USA). The nucleotide sequence was sequenced by Taihe Biotechnology Co., Ltd. (Beijing, China).

The recombinant plasmid, named as pET21a-Algpt, was transformed into *Escherichia coli* BL21 (DE3) and the *E. coli* were spread on LA media with 100 μg/mL ampicillin. Single colonies of transformants were picked and verified through enzymatic digestion and sequencing. The correct transformants carrying the pET21a-Algpt were incubated in an LB medium containing ampicillin (100 μg/mL) at 37 °C until OD_600_ reached 0.6–0.8. In order to induce the expression of Algpt, 0.5 mM isopropyl-β-D-thiogalactoside (IPTG) was added and the incubation was continued for 20 h at 20 °C. The cells were collected via centrifugation and subsequently suspended in lysis buffer (10 mM PBS, pH 7.4), and then subjected to sonication. The supernatant of cell lysis was purified and concentrated through Ni–NTA resin (Millipore, Burlington, MA, USA), equilibrated with binding buffer (50 mM NaH_2_PO_4_, 300 mM NaCl, 10 mM imidazole, pH 8.0). The other needless protein was washed off with washing buffer (50 mM NaH_2_PO_4_, 300 mM NaCl, 20 mM imidazole, pH 8.0), and the recombinant alginate lyase was eluted with elution buffer (50 mM NaH_2_PO_4_, 300 mM NaCl, 250 mM imidazole, pH 8.0). The target protein was further analysed using sodium dodecyl sulfate polyacrylamide gel electrophoresis (SDS-PAGE) [20]. Protein concentrations were determined using a BCA protein quantitative analysis kit (Beyotime Biotechnology, Shanghai, China).

### 4.5. Enzymatic Activity Assay

As for enzymatic activity measurement, 0.1 mL of diluted enzyme solution was mixed with 1.9 mL of 20 mM Tris-HCl buffer (pH 8.0) containing 5 g/L sodium alginate. Then, the mixture was cultured at 30 °C (unless otherwise specified) for 20 min. After incubation, the mixture was placed in a boiling water bath for 10 min to stop the enzymatic hydrolysis reaction. The enzymatic activity was determined by measuring absorbance at 235 nm (A235) on a TU-1901 (PERSEE, Beijing, China). One unit (U) was defined as the amount of enzyme required for each 0.1 increase in A235 value per minute.

### 4.6. Biochemical Characterization of the Recombinant Alginate Lyase

The effect of temperature on Algpt activity was measured at different temperatures ranging from 25 to 50 °C for 20 min. The thermal stability of Algpt was then denoted by detecting its remnant activity after pre-incubation at 50 °C for 1–3 h. The effect of pH on Algpt activity was determined at a series of pH values ranging from 4.0 to 9.0 with an interval of 1 pH unit for 20 min. The pH of the reaction system was adjusted by 50 mM buffer systems of sodium acetate (pH 4.0–6.0), NaH_2_PO_4_–Na_2_HPO_4_ (pH 6.0–8.0), and Tris-HCl (pH 7.0–9.0).

The effects of NaCl on enzymatic activity were measured at different concentrations of NaCl (100–700 mM) as described in 4.5. The enzymatic activity without NaCl (control) was defined as 100%.

### 4.7. Detection and Analysis of the Enzymatic Depolymerized Products

For the TLC assay, the samples collected at different reaction times were spotted on a silica gel 60 F254 plate (Merck, Darmstadt, Germany) and subsequently spread with a mobile solvent that consisted of 1-butanol/formic acid/water (4:6:1 v:v:v). Then the plate was sprayed with 10% (*v/v*) sulfuric acid in ethanol and heated at 110 °C for 10 min.

ESI–MS was used to further analyse the type and ratio of AOS with different DPs in enzymatic hydrolysate. The reaction solution was added with triploid anhydrous ethanol to remove polysaccharide and other macromolecular components. After centrifugation, the supernatant was filtered using a 0.22 μm filter membrane. An LCQ Fleet liquid and ion trap mass spectrometer (Thermo Fisher Scientific, Waltham, MA, USA) was used in electrospray negative ion mode for direct sample analysis. Instrument parameters were set as follows: capillary voltage, 4.00 kV; atomizer pressure, 3.4 bar; gas flow rate, 10.0 L/min; reaction temperature, 180 °C.

## Figures and Tables

**Table 1:** The comparison of enzymatic properties between Algpt and other alginate lyases from different microorganisms.

Alginate Lyase	Source	Temperature ^1^	pH	NaCl (mM)	Family	Reference
Algb	*Vibrio* sp. W13	30 °C, 20 °C–30 °C	8.0, 7.0–9.0	300, 200–600	PL-7	[33]
Alg2A	*Flavobacterium* sp. S20	40 °C, 40 °C–50 °C	8.5, 8.0–9.5	N.D. ^2^	PL-7	[34]
AlyPL6	*Pedobacter hainanensis* NJ-02	45 °C, 30 °C–50 °C	9.0, 9.0–10.0	N.D.	PL-6	[17]
OUC-ScCD6	*Streptomyces ecolicolor* A3(2)	50 °C, 30 °C–60 °C	9.0, 9.0–10.0	N.D.	PL-6	[35]
FsAlyPL6	*Flammeovirga* sp. NJ-04	45 °C, 40 °C–50 °C	9.0, 8.0–9.0	N.D.	PL-6	[29]
BcelPL6	*Bacteroides cellulosilyticus* CRE21	30 °C	7.5, 7.5–8.0	150, 150–250	PL-6	[18]
AlgF	*Vibrio splendidus* OU02	30 °C, 25 °C–35 °C	7.5, 7.0–8.0	200, 50–200	PL-6	[36]
Algpt	*Paenibacillus* sp. LJ-23	45 °C, 30 °C–50 °C	7.0, 6.0–9.0	0–700	PL-6	This study

^1^ Optimum condition of factor for alginate lyase, the range of factors that within which alginate lyase activity was retained at over 80%. ^2^ Not determined.

## Data Availability

The data presented in this study are available on reasonable request from the corresponding author.
